# The Role of Membrane Bound Complement Regulatory Proteins in Tumor Development and Cancer Immunotherapy

**DOI:** 10.3389/fimmu.2019.01074

**Published:** 2019-05-21

**Authors:** Anne Geller, Jun Yan

**Affiliations:** ^1^Department of Microbiology and Immunology, University of Louisville School of Medicine, Louisville, KY, United States; ^2^Immuno-Oncology Program, Department of Medicine, The James Graham Brown Cancer Center, University of Louisville School of Medicine, Louisville, KY, United States

**Keywords:** mCRP, complement cascade, oncology, immunotherapy, combination therapy

## Abstract

It has long been understood that the control and surveillance of tumors within the body involves an intricate dance between the adaptive and innate immune systems. At the center of the interplay between the adaptive and innate immune response sits the complement system—an evolutionarily ancient response that aids in the destruction of microorganisms and damaged cells, including cancer cells. Membrane-bound complement regulatory proteins (mCRPs), such as CD46, CD55, and CD59, are expressed throughout the body in order to prevent over-activation of the complement system. These mCRPs act as a double-edged sword however, as they can also over-regulate the complement system to the extent that it is no longer effective at eliminating cancerous cells. Recent studies are now indicating that mCRPs may function as a biomarker of a malignant transformation in numerous cancer types, and further, are being shown to interfere with anti-tumor treatments. This highlights the critical roles that therapeutic blockade of mCRPs can play in cancer treatment. Furthermore, with the complement system having the ability to both directly and indirectly control adaptive T-cell responses, the use of a combinatorial approach of complement-related therapy along with other T-cell activating therapies becomes a logical approach to treatment. This review will highlight the biomarker-related role that mCRP expression may have in the classification of tumor phenotype and predicted response to different anti-cancer treatments in the context of an emerging understanding that complement activation within the Tumor Microenvironment (TME) is actually harmful for tumor control. We will discuss what is known about complement activation and mCRPs relating to cancer and immunotherapy, and will examine the potential for combinatorial approaches of anti-mCRP therapy with other anti-tumor therapies, especially checkpoint inhibitors such as anti PD-1 and PD-L1 monoclonal antibodies (mAbs). Overall, mCRPs play an essential role in the immune response to tumors, and understanding their role in the immune response, particularly in modulating currently used cancer therapeutics may lead to better clinical outcomes in patients with diverse cancer types.

## Introduction

The complement system is an evolutionarily primordial component of the innate immune response that functions through a series of over 30 coordinated cascading proteins and zymogens to protect the body from invading pathogens (1). The proteins of the complement system can be found both in the plasma and as inactive precursors on the surface of cells within the body, and when activated by foreign pathogens lead to opsonization and eventual lysis of foreign cells. Though complement is an essential part of the immune response against microbes, the complement system also plays crucial roles in maintaining homeostasis through such mechanisms as the removal of apoptotic cells, the regulation of coagulation, angiogenesis, and lipid metabolism and, importantly, the surveillance of neoplastic cells (2–6). Furthermore, as in all cases of homeostasis, just as the complement pathway can be activated, it too must be kept under the tight control of negative regulators so as to prevent excessive damage to self-tissues. Atypical hemolytic uremic syndrome (aHUS), C3 glomerulopathy (C3g), and paroxysmal hemoglobinuria (PNH) are all examples of serious pathological clinical conditions resulting from inadequate control of the complement system, highlighting the importance of complement regulation (7). Membrane-bound Complement Regulatory Proteins (mCRPs) are one such factor that exerts tight regulatory functions on the complement system thus protecting the body from the deleterious effects of overactive complement. While the regulation of the complement system is becoming relatively well-studied, the relationship between the regulation of the complement system and the surveillance of neoplastic cells is not well-understood, mainly due to the fact that there exists a dichotomy in the understanding of the relationship between tumorigenesis and complement. On one hand it is thought that complement is a necessary check to neoplastic cells, and thus the expression of mCRPs allows tumor cells to proliferate unchecked, while on the other hand it has been observed that chronic inflammation can promote carcinogenesis indicating that, to a certain extent, mCRP expression may be protective against tumor growth. In this review we will discuss what is known about the role of mCRPs in regulating tumor growth, how their expression may be used as a biomarker to assess malignancy in certain cases, and how this evolving knowledge of mCRPs can be combined with the growing arsenal of immunotherapy to create improved outcomes for cancer patients.

## The Complement System

The complement system recognizes foreign pathogens and self-cells expressing aberrant surface molecules indicative of damage through three converging pathways: the classical, lectin, and alternative pathways. The classical pathway is activated by immune complexes of antigens and antibodies. The C1 complex, consisting of C1q and two serine proteases, C1r and C1s, circulates in the serum in an inactive state. When the inactive C1q component binds to the Fc region of IgM or IgG complexed with antigen, a conformational change occurs which results in the activation of C1r and C1s (8). Activated C1s will cleave C4 into C4a and C4b, and C2 into C2a and C2b. Subunits C4b and C2a will then bind non-covalently resulting in the creation of C4bC2a, a C3 convertase enzyme complex (9, 10). In the lectin pathway, pattern-recognizing mannose-binding lectins (MBLs) and ficolins bind to carbohydrate ligands, such as mannose, present on the surface of pathogens and together with MBL-associated serine protease 2 (MASP2) forms a C1-like complex that cleaves C4 and C2 resulting in a C3 convertase C2aC4b. Finally, in the alternative pathway, stimulation occurs through spontaneous hydrolysis of C3 or the sensing of a foreign surface structure. In this process, hydrolyzed plasma C3 [C3(H20)] and factor B bind, with the help of Factor D, create C3(H_2_0)Bb. The C3(H_2_0)Bb complex will cleave plasma C3, resulting in C3b, which will bind to cell surfaces and to Bb, resulting in C3Bb. C3Bb is the functional C3 convertase of the alternative pathway.

C3 is the point of convergence between the three complement pathways, where despite different mechanisms of activation, the effector result becomes synonymous. The cleavage of C3 results in the production of C3a, a major anaphylatoxin, and C3b, an important molecule known as an opsonin which is able to coat the surface of antigens thereby marking them for phagocytosis by circulating macrophages. C3 convertase will also create a C5 convertase by binding to available C3b molecules. C5 convertase cleaves C5 to create C5b, which then binds with C6, C7, C8, and multiple C9 to form the C5b-9 complex. This complex is also known as the Membrane Attack Complex (MAC) and will be deposited into the lipid bilayer of cells eventually resulting in membrane destruction and cellular lysis.

While the MAC is an important effector arm of the complement system, there are several pathogens which are resistant to MAC lysis due to such structures as the cell wall found in gram-positive bacteria (11) or the generation of microbial complement inhibitors, such as the streptococcal inhibitor of complement (SIC) which is capable of preventing MAC formation through interference with the C5b-C7 and C5b-C8 complexes (11, 12). For these reasons the pro-inflammatory signaling and the phagocytic functions of complement are just as, if not more important than the direct effects of cell lysis. During amplification of the complement system, C3a and C5a are released in a constant stream, which functions through G-protein coupled receptors (GPCRs) C3aR and C5aR, respectively, to signal as powerful chemo-attractants for neutrophils, monocytes, eosinophils, mast cells and macrophages (13–17). Furthermore, opsonins C3b and C4b aid in phagocytosis by binding to proteins and polysaccharides on microbial and foreign surfaces and receptors, such as CR1 expressed on phagocytes. With regards to cancer, both the chemoattractant and opsonization properties of complement activation have serious implications for the immune composition of the tumor microenvironment.

### The Complement System and It's Interaction With Tumor Cells

The expression of various surface markers on tumor cells has been found to activate all three pathways of the complement system. The classical pathway has been found to be activated by specific molecules expressed on the surface of tumorigenic cells. The general mechanism involves the recognition of post-transcriptionally modified tumor-specific antigens by natural IgM, which unlike IgG, is capable of binding C1q with only a single molecule (18). Natural IgM is IgM produced without prior antigenic stimulation and without the intervention of adaptive immune responses to an antigen. It exists in low levels to help the body maintain homeostasis and to recognize cells that have been invaded by a foreign pathogen, and senescent, apoptotic, precancerous, and cancerous cells (19–22). In one such example, the expression of gangliosides GD3 and GD2 expressed on the surface of melanoma and neuroblastoma cells can be recognized by natural IgM antibodies in the sera of a limited number of healthy individuals, resulting in complement mediated cell lysis (23, 24). In another study, an antibody, SC-1, was isolated from a patient with signet ring cell carcinoma of the stomach and found to be reactive to all diffuse-type stomach cancer cells, and around 20% of intestinal-type adenocarcinomas. Upon reaction, the antibodies were found to induce apoptosis of the cancerous cells through a complement mediated pathway, and in clinical studies, SC-1 was able to induce regression of primary stomach cancers (25–28).

The lectin pathway has been shown to be activated in numerous glioma cell lines, where glioma cells expressing high levels of mannose-glycoproteins are easily bound by MBL, resulting in C3 and C4 activation (29). Finally, in cancers driven by virus-dependent transformation, such as EBV-infected B lymphoblastic cell lines and HIV infected T-cell lines, the alternative pathway is quickly able to recognize aberrantly expressed viral carbohydrate particles on the surface of infected cells, resulting in complement activation (30–33).

Overall, while complement is shown to be activated by tumor cells, whether this activation is actually beneficial to tumor eradication has come under intense scrutiny. A simple explanation for this is that while to a certain extent inflammation is beneficial for the control of neoplastic cells, prolonged inflammation, which could be caused by activated complement cascades, actually promotes oncogenesis (34). This theory is supported by the clinical example of the link between intraprostatic inflammatory lesions, prostatic intra-epithelial neoplasia, and cancer (35). The association of an inflammatory state and cancer is further supported by evidence that non-steroidal anti-inflammatory drug use is associated with reduced incidence of colorectal and gastric cancers (36, 37).

The first correlation between the complement cascade and increased tumor growth came from a study by Markiewski et al. where cervical tumors were transplanted into C3-deficient mice and wild-type (WT) mice. In this study tumors grew faster in WT mice as compared to C3-deficient mice, indicating that C3 may promote tumor growth. They then used the same experimental design in C5a receptor-deficient mice and found that C5a also aids in tumor growth by binding to C5a expressed on myeloid-derived suppressor cells (MDSCs). Binding to MDSCs prompted granulocytic/neutrophil-like MDSCs to migrate to the tumor, and also increased ROS and reactive nitrogen species production in monocytic MDSCs, both of which resulted in stronger suppressive MDSC effects on T-cells (38, 39). Bulla et al. performed a similar study where they found that as compared to WT mice, C1q deficient mice bearing syngeneic B16 melanoma had a slower tumor growth, fewer lung metastases, and prolonged survival. It has also been noted that the expression of complement and complement reactive proteins is present in measurable quantities in many malignant cancers (40). A final example of the deleterious effects of complement on the control of oncogenesis comes from a study by Wang et al. which showed that C3, acting through C5aR and C3aR on the surface of CD8+ tumor-infiltrating lymphocytes (TILs), is able to constitutively suppress IL-10 production. This data ultimately showed that complement activation in the tumor microenvironment suppresses the anti-tumor effects of CD8+ TILs (41, 42).

### mCRPs

As is the case in any homeostatic process, there are several regulatory mechanisms in place to ensure that the complement system does not become over activated, thus causing harm to self-tissues. There are several soluble regulatory proteins such as C1 inhibitor, C4b binding protein, and factors H, B, D, and I. In addition, mCRPs are another control mechanism that includes CD35 (Complement receptor 1, CR1), CD46 (membrane cofactor protein, MCP), CD55 (decay acceleration factor, DAF,), and CD59 (protectin) (43, 44). In fact, complement regulatory proteins are expressed on every cell in the body (45), though the expression of these mCRPs varies across tissue type. It can be hypothesized that because different tissues face different immune interactions, the mCRP expression across tissue type is variable (46).

### CD35

CD35 is primarily expressed on erythrocytes, lymphocytes, phagocytes and dendritic cells, with rare expression on tumor cells (47, 48). It functions as a cofactor for the cleavage of C3b into iC3b (49). Additionally, CD35 binds to C4b and promotes the degradation of C4b into C4c and C4d. Importantly, CD35 is also involved in accelerating the decay of C3/C5 convertases, resulting in an inhibition of complement activation at the level of the C3 cascade. Previously it had been shown that CD35 expression could be found in follicular dendritic cell tumors, malignant endometrial tissue, and leukemic blasts (44, 50, 51). More recently, studies have also linked the expression of CD35 on both tumor and on immune cells to a susceptibility for gallbladder cancer (52), advanced clinical stage and poor overall survival in patients diagnosed with nasopharyngeal cancer (53).

## The Function of CD46

**Figure 1:**
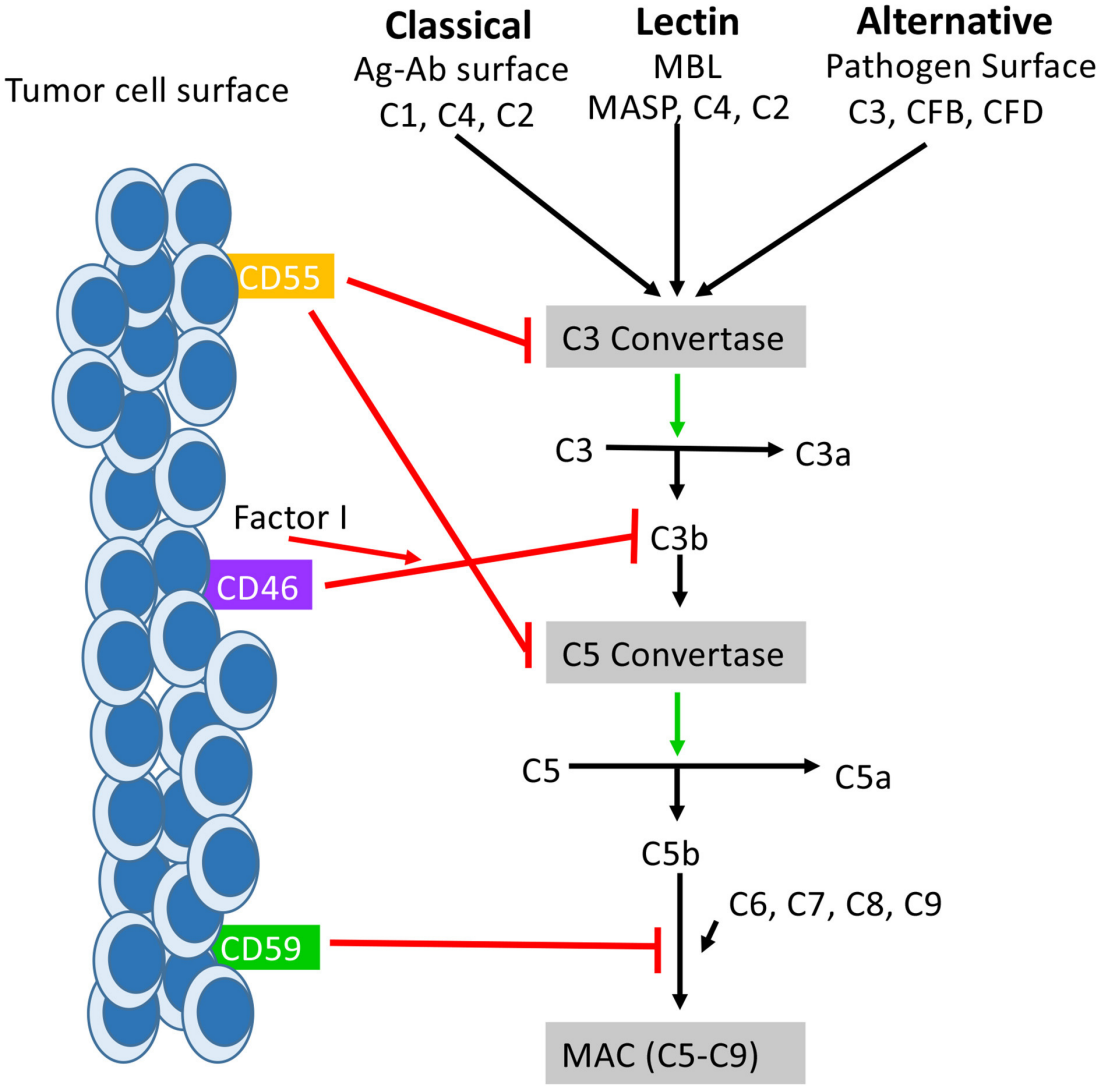
How mCRPs regulate the complement cascade: mCRPs CD55, CD46, and CD59 exert a regulatory influence on the complement cascade to prevent complement from becoming overly activated. CD55, CD46, and CD59 are known to exert control on all three pathways of complement activation. CD55, also known as DAF, accelerates the decay of the C3 convertases (C4bC2a and C3bBb) and consequently the C5 convertases into constituent elements and prevents re-association (55). The outcome is destabilization of the C3 and C5 convertases which results in decreased anaphylatoxin (C3a,C4a, C5a) formation, decreased opsonin formation (C3c and iC3b), and prevention of MAC formation. CD46 functions as a cofactor for Factor I in the cleavage of C3b and C4b (not shown), leading to inactivation of both (56). CD59 prevents the polymerization of C9 and insertion of additional C9 molecules into the C5b-9 complex (57). It also directly interferes with pore formation of C5b-8, resulting in inhibition of MAC formation. While the distribution of CD55, Cd46, and CD59 is varied across tissues of the body, they are all found expressed on the surface of various tumor cells where they serve as biomarker for tumor formation.

**Figure 2:**
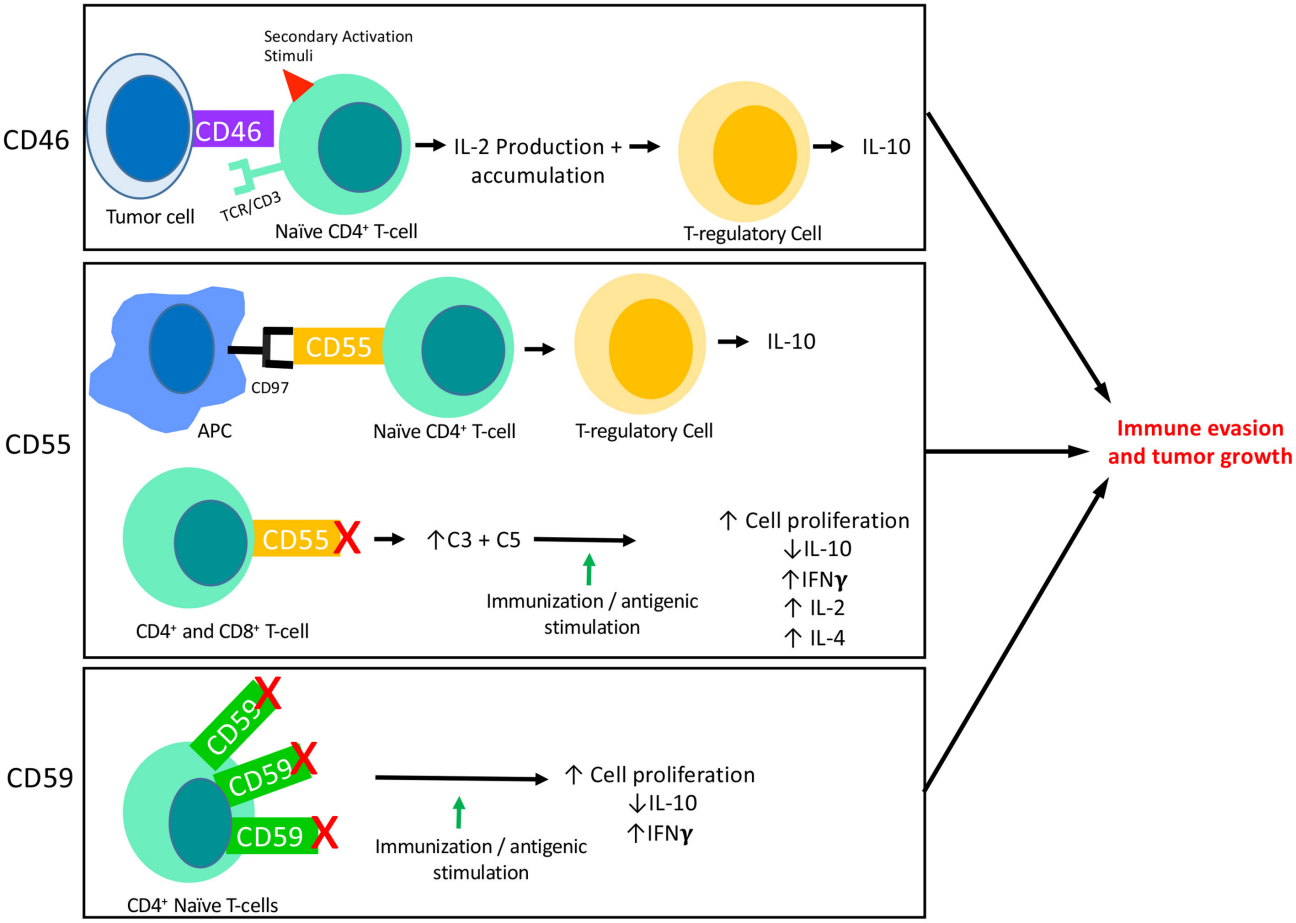
The interaction of mCRPs with the adaptive immune response: CD46, CD55, and CD59 all have known interactions with the adaptive immune response. This figure summarizes what is currently known about each of their interactions with adaptive responses, specifically T-cell responses. CD46 is known to be expressed on the surface of tumor cells and its binding to a naïve CD4+ T-cell in the presence of a secondary activation stimuli results in IFNγ and IL-2 production. Though initially immuno-stimulatory, as IL-2 accumulates it causes activated CD4+ T-cells to undergo a transformation into a Th1 Regulatory cell that produces high levels of IL-10. Two important aspects of CD55 activity are shown here. First, CD55 on the surface of T-cells are known to interact with CD97 displayed on the surface of Antigen Presenting Cells (APCs). This interaction leads to a shift in T-cell functionality, resulting in T-cells that function like TRegs and produce IL-10. The blockade of CD55 on the surface of T-cells has also revealed the immunosuppressive function of CD55. When CD55 is blocked on both CD4+ and CD8+ naïve T-cells followed by immune stimulation (*in vitro*) or immunization (*in vivo*), T-cells are shown to proliferate and to produce increased IFNγ, IL-2, and IL-4 and decreased IL-10 as compared to cells or animals that were untreated. This effect appears to be dependent on the increased levels of C3 and C5 present due to blocked functionality of CD55. In certain circumstances, CD59 is found to be overexpressed on CD4+ T-cells which results in downregulation of CD4+ activity. Accordingly, blockade of CD59 results in enhanced T cell responses consisting of increased cell proliferation, decreased IL-10 production and increased IFNγ production.

CD46, CD55, and CD59 are the mCRPs whose function most relates to tumors. Together, these surface proteins are also known to inhibit complement responses, and of late have also been a focus of research related to human malignancy. CD46 is a transmembrane glycoprotein that is expressed on all nucleated cells, and like CD35, functions to protect excessive complement activation by acting as a cofactor in the proteolytic cleavage of C3b and C4b, mediated by Factor I (54) (Figure 1). Though CD46 may initially have been thought to primarily function as a mCRP, CD46 has also been found to have functionality in mediating immune responses. For example, CD46 has been found to act through distinct mechanisms to regulate different T-cell subsets during an immune response, where CD46 actually acts as a costimulatory molecule for T-cells. Specifically, the binding of CD46 on CD4+ T cells has been found to result in an initial proliferation and activation of T helper type 1 cells (T_H_1 cells), with a characteristic production of Interferon γ (IFN γ) (58). However, a simultaneous expansion of effector cells leads to an accumulation of interleukin 2 (IL-2), which provides a switch signal for CD4+ T-cells to take on a T regulatory (Treg) phenotype. CD4+ cells then begin producing IL-10 in order to control the expanding immune response (Figure 2). When CD46 is dysregulated, this switch to a Treg phenotype does not occur, which clinically has been related to chronic inflammatory diseases such as relapsing and remitting Multiple Sclerosis (MS) (59), asthma (60), and Rheumatoid Arthritis (RA) (61). Additionally, as discussed above, such a chronic inflammatory state can allow pre-metastatic cells to thrive (62). CD46 activation on γδ T-cells has also been shown to directly suppress their IFNγ and TNFα production, which can further lead to a pro-tumor environment (61, 63, 64). Together this data suggests a temporally and spatially regulated role of CD46 in adaptive immune responses, which also serves as an important indication that the complement cascade is capable of exerting a driving influence on adaptive T cell responses during anti-tumor responses.

In terms of the regulation of CD46, it has been shown that CD46 is highly glycosylated, and that CD3 stimulation alters the O-glycosylation of CD46 in activated T-cells, resulting in decreased CD46 processing and T-cell singling, which ultimately leads to a T-reg phenotype characterized by the dominance of IL-10. Nuclear factor κB (NF-κB) has also been shown to regulate CD46 expression, where activation of NF-κB is critical for CD46 expression (65).

CD28, which is a receptor on T-cells that provides a secondary activation signal for T-cells in conjunction with the primary TCR signal (66), has also been identified to have an important role in regulating CD46 signaling. Not only has CD28 been shown to control CD46 expression on activated T-cells, but Charron et al. also showed that the engagement of CD28 and CD46 mediates T-cell responses. In regards to the IFNγ:IL-10 production ratio, as compared to CD28 stimulation alone, CD28/CD46 co-stimulation was shown to promote regulatory function, while compared to CD46 activation alone, CD28/CD46 co-stimulation was shown to decrease regulatory function (67). Together this data indicates the intricate role of CD28 in regulating CD46, and the important cytokine-related role that these two may play in tumor specific adaptive responses.

## CD46 as a Biomarker for Cancer

Combining this data of adaptive T cell responses, which seem to be anti-tumor in certain circumstances, and pro-tumor in others, with the fact that it is still not unanimously agreed upon whether complement expression is beneficial to tumor defense, it seems the role of mCRP CD46 is not as clear cut as originally hypothesized. For this reason, investigators have sought to characterize CD46 expression on various tumors, with the potential goal of using CD46 as a biomarker to predict immune response and patient outcome. In ovarian cancer for example, CD46 expression was linked to shorter revival-free time, defined as the time from the primary surgical treatment until the time of diagnosis of a recurrent tumor or death, and an overall less favorable outcome (68). Similar findings have also been found in breast cancer cases, where CD46 expression and involvement of lymph nodes represent independent risk factors for disease-free survival, and CD46 expression was found to be linked to less favorable diagnoses (69). Other cancers found to express higher levels of CD46 than adjacent normal tissues, which also relates to a worse clinical prognosis, include hepatocellular carcinoma (HCC), colon cancer, and Multiple Myeloma (70–72).

## CD55 and CD97

CD55 also functions as an inhibitor of both the classical and the alternative pathway of complement activation where, unlike CD35 and CD46 which act in a proteolytic fashion, it accelerates the decay of C3 and C5 convertases. CD55 does this by inducing a rapid dissociation of C2a or Bb catalytic subunit present in convertases on the cell surface (73) (Figure 1). Like CD46, CD55 has also been shown to have important effects on the adaptive immune response, where CD55 has been linked to the suppression of adaptive immune responses *in vivo*. For example, in mice lacking the Daf1 gene, which encodes the murine homolog of human DAF, CD4+ T-cells were found to produce more IFNγ and IL-2 and less IL-10 in response to active immunization (74). Other investigators have found that during primary T-cell activation, the absence of CD55 on APCs and T-cells enhances the proliferation and leads to enhanced effector cell frequency (75). In a model of CD8+ T-cell immune responses to lymphocytic choriomeningitis virus infection, mice lacking Daf had increased CD8+ T-cell expansion in spleen and lymph nodes, and an increased number of antigen-specific CD8+ T-cell, which resulted in faster infection clearance (76). These effects were ultimately linked to the presence of increased complement proteins due to a lack of CD55 expression and not CD55 itself, as knocking out C3 expression in CD55^−/−^ mice restored normal responses (77). Overall however, the expression of CD55 decreases complement mediated cell lysis in tumors, and a lack of CD55 increases the overall inflammatory response (78–81) (Figure 2). In an effort to explain the observation of enhanced T-cell responses in DAF^−/−^ mice, another group investigated whether CD55 expression influences the stimulatory power of antigen presenting cells (APCs). In this study APCs from DAF^−/−^ mice treated with an inflammatory stimuli elicited more potent T-cell responses in a complement dependent manner, and also had decreased PD-L1 and increased CD40 on the cell surface (82). Natural killer (NK) cell responses have also been shown to be inhibited by CD55 (83).

The regulation of CD55 is also versatile as the synthesis and expression of CD55 on tumor cells has been shown to be influenced by IL-1α, IL-1β, IL-4, EGF, TNF-α, IFNγ, and Prostaglandin E2 (84–88).

Another important factor regarding CD55 relates to CD97, an EGF-TM7 receptor expressed primarily on monocytes and granulocytes that acts as a natural ligand for CD55 (89). Together, this complex acts as a T cell receptor co-stimulatory protein complex (90). A study by Capasso et al. showed that direct CD55 engagement with CD97 and co-stimulation with CD3 results in T-cell activation involving increased T-cell proliferation, IL-10, and GM-CSF production, and expression of activation markers CD69 and CD25 (77). Importantly, the naïve T cells that are stimulated in response to CD55 and CD97 binding are shown to produce cells that behave like Tregs, which would promote tumor progression if expressed in the TME (91).

### CD55 as a Biomarker

It is not entirely clear whether CD55 is expressed by tumors to help defend against the deleterious effects of complement activation, or whether CD55 expression on tumors is more functionally related to its role as a ligand for CD97 in T-cell activation. Either way, it is clear that CD55 is not only present in cancer tissues, but also that it plays an important permissive role in the progression of tumorigenesis. In many cases, for example in colon cancer, CD55 serves as a marker of tumor aggression and decreased 7-year survival (92). In the setting of breast cancer, it was found that cells expressing high CD55 levels were more resistant to apoptotic stimuli, have a higher growth rate, and in human cancer, are an independent prognostic factor for recurrence (93). Other cancers that show high expression of CD55 and worse clinical prognoses as a result include prostate cancer, ovarian cancer, AML, CML, ALL, gastric carcinoma, and cervical cancer (94–99).

Overexpression of CD55 in Barrett's esophagus has also been associated with esophageal adenocarcinoma risk (100). Interestingly, in this instance it appears that rather than CD55 being a marker of tumor cells, CD55 expression instead lends to a microenvironment that is favorable for a malignant transformation. In some sense, it leads to a question of the chicken or the egg—is CD55 expression upregulated which then leads to an ability for tumor cells to proliferate unchecked by complement and a microenvironment permissive to tumor growth, or do tumor cells form, and then as a secondary defense mechanism express CD55 to protect against complement destruction. Such a clarification has not been made, though it is important as the distinction could indicate clinical treatment using mAbs to be more appropriate for premalignant vs. malignant states. This distinction may also be helpful in understanding the seemingly dual role that complement plays in tumor cells. On one hand, the expression of CD55, which results in a downregulation of complement activity may be a protective mechanism to the inflammatory milieu of a premalignant state, aiming to protection against further inflammatory stimuli and a malignant transformation. Alternatively, the expression of CD55 could prevent complement mediated killing of premalignant cells, resulting in decreased control of tumor growth.

### CD97 as a Biomarker

With CD55 showing such impressive potential as a biomarker for malignant states and prognosis, it is logical that CD97, which binds to CD55 and controls adaptive T cell responses, would also have utility as a biomarker. In intrahepatic cholangiocarcinoma, for example, CD97 and CD55 together were associated with histological grade, and increased biliary soluble levels of CD97 specifically was an independent risk factor for patient survival (101). CD97 and CD55 are also upregulated in pancreatic cancers, and are associated with lymph node involvement, metastasis, and vascular invasion. Wu et al. identified CD97 and CD55 to be upregulated in human gallbladder carcinoma (102), and Mustafa et al. showed that CD97 is a specific biomarker for dedifferentiated oral squamous cell carcinoma and that it accurately predicts grading and staging of disease (103, 104). Rectal adenocarcinoma, cervical squamous cell carcinoma, medullary thyroid carcinoma, and gastric carcinoma were also shown to exhibit similar trends (99, 105–107).

In a study by Steinert et al. histopathological staining showed that in human colorectal cancer, normal colorectal epithelium did not stain for CD 97, while 75% of carcinomas did express CD97. Further, the most significant staining of CD97 occurred at the invasion front. A dispersed pattern of CD97 was correlated with a poorer clinical stage as compared to those tumors that expressed CD97 in a uniformed pattern (108). This information indicates that CD97 is involved in tumor migration, invasion and differentiation (109). Others hypothesize that CD97 and CD55 may facilitate the adhesion of cells to surrounding surfaces, facilitating metastasis (103). Thus, CD97 may not only serve as a biomarker of tumor aggressiveness and early metastasis, but it may also serve as an effective therapeutic target.

## CD59

CD59 inhibits the polymerization of C9 and it's binding to C5b-8 through competitive inhibition of an epitope on C8, resulting in inhibition of MAC assembly and cell lysis (110–112) (Figure 1). CD59 plays a critical role in the protection of self-tissues and is widely expressed on most tissues in the human body including erythrocytes, monocytes, heart, spleen, liver, and kidney (113). The protective effects of CD59 are so important that pathogenically low levels of CD59 are associated with autoimmune diseases such as diabetes, multiple sclerosis, and chronic hemolysis (114–116). Like CD46 and CD55, CD59 is also involved in T-cell responses, where CD59 is upregulated on CD4+ T cells and leads to down regulation of CD4+ activity. Accordingly, blockade of CD59 results in enhanced T cell responses (Figure 2) (117).

### CD59 as a Biomarker

Predictably, CD59 also has been shown to have a biomarker related function for various tumors. Increased expression of CD59 is associated with reduced survival in colorectal cancer patients (118), and with decreased overall survival and progression-free survival in patients with diffuse large B cell lymphoma and adenocarcinomas of the prostate (119, 120). The opposite is true in breast tumors however, where loss of CD59 expression in breast tumors correlates with poor patient survival. The authors of this finding hypothesize that the loss of CD59 may provide a “selective advantage” for breast cancers, which results in more invasive tumors (121). This may also relate to the findings regarding the potentially deleterious role that complement activation can play in tumors.

### mCRPs and Tumor Therapy

Because of the great deal of data showing that CD46, CD55, and CD59 expression are linked to worse clinical outcomes, and are in some cases highly specific for tumor cells, many approaches to block mCRP expression on tumor cells have been studied. The first and perhaps most studied of these approaches is neutralizing mAbs. Overall these have shown effective enhancement of tumor cell susceptibility to complement mediating killing in a wide range of tumor types (122). For example, neutralization of CD55 has led to increased complement activation and complement-mediated killing in Burkitt lymphpoma (81), leukemia (123), melanoma (124), and breast cancer (125). The same can be said for the blockade of CD59 with neutralizing mAb and neuroblastoma (126), leukemia, breast (127), ovarian (128), and renal cancers (129). Small interfering RNAs (siRNAs) (130) and anti-sense phosphorothioate oligonucelotides (S-ODNs) (131) have also been successfully used to downregulate mCRP expression in tumors, which in many cases leads to mitigation of tumor burden.

Recently, neutralizing mAbs have also been employed concomitantly with chemotherapeutic drugs to achieve improved outcomes, especially in patients who are non-responsive to initial chemotherapeutic treatment, often due to an initial overexpression of mCRP. CD20-postitive Burkitt lymphoma Raji cells and primary CLL cells are generally resistant to the complement-dependent cytotoxicity induced by rituximab treatment. Mamidi et al. and Weiguo et al. independently showed that inhibition of mCRP expression, specifically CD59 (132), sensitizes cancerous leukemia cells to complement attack, resulting in enhanced effectiveness of rituximab (122). Similarly, the use of mAbs blocking CD55 and CD59 in addition to Rituximab treatment leads to increased tumor toxicity in non-Hodgkin's lymphoma (133). Results have shown that in Herceptin treatment for non-small cell lung cancer (NSCLC), neutralization of CD55 and CD59 results in markedly increased Herceptin-mediated complement cytotoxicity. Even more interesting, this study showed that overexpression of mRPs on tumor cells is likely largely responsible for Herceptin resistance in NSCLC (134). CD55 and CD59 expression were also correlated with the protection of HER2-overexpressing breast cancer and uterine serous carcinoma cells from trastuzumab-induced complement dependent cytotoxicity (135, 136). CD55 has been identified as a signaling protein responsible for self-renewal and therapeutic resistance to cisplatin in endometroid tumors, and blockade of CD55 using saracatinib sensitizes chemo-resistant cells to cisplatin (137). A human CD59 inhibitor has been shown to enhance complement dependent cytotoxicity of ofatumumab against rituximab-resistant B-cell Lymphoma cells and CLL (138). In a slightly different approach, Su et al. used a model of prostatic cancer, where CD46 was found to be overexpressed in primary tumor tissue in metastatic castration-resistant prostate cancer (mCRPC) but not on normal tissues, and was able to show excellent selective killing of cancer cells by using an antibody-drug conjugate (ADC) consisting of a tubulin inhibitor and a macropinocytosing anti-CD46 ADC. Their CD46 ADC caused regression and elimination of a mCRPC cell line xenograft, showing the efficacy of targeting CD46 in combination with a tubulin inhibitor as a means to treat cancer (139).

Though the inhibition of mCRPs has shown marked efficacy in harnessing the power of the complement cascade to control tumor growth, such therapies pose a threat of causing over activation of the complement cascade in normal tissues, as mCRPs are expressed on normal tissues ubiquitously throughout the body. As a result, a fear of non-specific mCRP blockade is the development of autoimmune-like disease, as could be expected considering the auto-immune diseases associated with genetic mutations of specific mCRPs (140). Despite these fears, there are several examples of anti mCRPs therapies being used both successfully and safely. For example in a study using both transgenic mice and macaques, the transient depletion of CD46 on the cell surface using a recombinant protein was not only able to sensitize tumors to complement mediated cytotoxicity, but was also shown to be safe and well-tolerated as defined by body weight and blood and chemistry analyses (141). In addition, to prevent possible off-target effects, efforts have been made to specifically deliver mCRP targeting therapeutics to the tumor site. One way to do this is to create antibodies with one F(ab) region specific to an mCRP and another F(ab) region with high affinity to a tumor-restricted antigen (43). In doing so, the potential side-effects of generalized anti-mCRP therapy can be extenuated. An example of the successful use of this strategy can be seen in a study by Gelderman et al. where the group designed a bispecific anti-CD55 and anti-Ep-CAM antibody that was able to precisely target and cause C3 deposition in cervical and colorectal carcinomas, which overexpress Ep-CAM (142, 143). These targeted therapies certainly provide an excellent approach to developing safer and more effective anti-cancer therapeutics, though more in-depth clinical studies are needed in order to further categorize potential toxicities of the various mCRP targeting drugs.

### A New Paradigm to Understand mCRP Expression

The successful use of mAbs directed against mCRPs suggests that targeting mCRP, especially when in combination with other chemotherapeutic drugs, does have valuable therapeutic value. While this may be true, it also remains the case that the role of complement in the TME is likely more deleterious to controlling tumor growth than it is helpful. The implication of this is that the expression of mCRPs in tumors should indicate less complement activation and therefore a better prognosis. The actuality is that mCRP expression by and large is indicative of increased TNM staging and worse overall patient survival. If put into the current paradigm of complement activation, where increased complement activation in a tumor results in enhanced tumor killing and thus increased patient survival, these ideas seem irreconcilable. In order to reconcile the role of mCRPs in tumor expression, we argue that mCRPs should be viewed as more of a biomarker of an aggressive tumor phenotype involving intense generalized inflammation rather than a functional measure of the amount of complement activation present in a given TME.

mCRPs have been found to be upregulated by inflammatory cytokines and in inflammatory conditions (82, 84, 85, 144), likely as a reactionary attempt to prevent pathological activation of complement. In a TME however, there are constant sources of inflammation and especially once tumor cells have escaped initial immune control, there is an intense infiltration of immune cells and activation of the complement cascade. As a result, mCRPs levels could continually rise in response to snowballing inflammation, despite being unable to fully control activation within the TME. As a result, mCRPs would be expressed most intensely in the most inflammatory environments, which as discussed above is an advantageous environment for tumor growth. In this paradigm, mCRPs would serve as an excellent biomarker for invasive and progressive disease though less of a therapeutic target. This understanding would also concurrently explain why both mCRP expression and complement activation in the TME are positively correlated with a worse overall patient survival.

### Complement and Checkpoint Inhibitor Therapy

Components of the complement cascade interact with adaptive immune responses in a myriad of ways. We have already discussed how almost all mCRPs are capable of downregulating T-cell activation and effector function through either complement-dependent or independent mechanisms. Further, with the recent success of PD-1 immune checkpoint blockade therapy, understanding the role that complement plays specifically in responses to therapy, and generally in responses of the adaptive immune system is of extreme importance.

We have already discussed that mice lacking CD55 mount more potent T cell responses upon stimulation than mice expressing CD55, which is requisite on C3 and C5aR signaling. Further, APCs in these CD55^−/−^ mice expressed decreased PD-L1 and increased CD40 after stimulation as compared to WT (82). Several other complement constituents have been found to regulate adaptive immune responses in similar ways. It has been established that T cells express C3a and C5a receptors, which when bound by ligand result in IL-10 production and suppression of tumor-specific CD8+ T cell mediated cytotoxicity in melanoma (145). C5a, which causes tissue damage by inducing pro-inflammatory cytokine and chemokine production, neutrophil migration and blood vessel permeability, has been shown to stimulate IL-10 and TGF-β production from myeloid cells which promotes Treg generation (146, 147). In another study, C5a was shown to induce PD-L1 expression on monocytes through the activation of ERK1/2 and JNK signaling pathways, showing yet another interaction of complement with T cell responses (148). Interestingly, PD-L1 blockade has also been shown to result in the production of massive amounts of C5a suggesting a synergistic relationship between the two (148, 149). Exploiting this relationship, one group examined the therapeutic efficacy of PD-1/PD-L1 blockade in C5aR^−/−^ mice, and found that C5a negatively regulates the efficacy of PD-1/PD-L1 blockade. Increased T-cell ratios and functions in the tumor tissue were observed when PD-1/PD-L1 agonists were used in combination with a C5aR antagonist (149). Clinically, dual blockade of PD-1 and C5a/C5aR has been shown to work synergistically to protect against NSCLC (150). It is hypothesized that these effects are due to C5a recruitment of MDSCs to the TME. PD-1/PD-L1 blockade cannot overcome the suppressive T cell activity of the MDSCs, so blockade of C5a thus reduces MDSCs in the TME and creates a niche more susceptible to PD-1 blockade (151). Finally, in a study where mass spectrometry was used to correlate baseline serum protein signatures with response to nivolumab in metastatic melanoma, patient survival could be partially predicted by the signature of proteins associated with acute phase reactant and elements of the complement cascade. In this study, the presence of complement pathway proteins was associated with poor outcomes in patients treated with checkpoint inhibition (152). Overall this data surprisingly points to the idea that the presence of complement proteins negatively regulates response to checkpoint inhibitor therapy.

Though clearly there exists ample data on the interaction of complement with checkpoint inhibitor therapy, there do not yet exist any studies linking the expression of mCRPs specifically to the efficacy of PD-1/PD-L1 blockade. Considering the widespread use of immunocheckpoint inhibitor therapy and the considerable interaction of mCRPs with T-cell activation, further understanding of how mCRPs impact PD-L1 expression, and impact PD-1/PD-L1 blockade therapy is of vital importance. It may be hypothesized that because complement products, for example C5a, negatively regulate PD-L1 responses, the use of neutralizing mAbs against mCRPs that increase C3a and C5a production in the TME such CD35, CD45, and CD55, would not be a useful combinatorial therapy. It could be argued however that because CD59 is acting on inhibition of the MAC formation which is more directly and immediately responsible for tumor killing, the blockade of CD59 in conjunction with immunocheckpoint inhibitor therapy may be useful. Additionally, because mCRPs have been shown to be a specific biomarker for many cancer types, therapies that take advantage of the capability of mCRPs to identify malignantly transformed cells in order to deliver immunocheckpoint inhibitors directly to a tumor tissue, while at the same time sparing normal tissue, could be extremely useful and lead to even better clinical outcomes in cancer patients treated with these regimens. Realistically, the same is true of almost any chemotherapeutic drug; mCRPs could be used to identify cancerous cells, and therapies could be designed to traffic to areas strongly exhibiting mCRPs or specific isoforms indicative of tumorigenesis depending on specific tumor type. Ultimately, more research is needed on the interaction of mCRPs and the growing arsenal of immunocheckpoint inhibitor therapies.

## Conclusion

mCRPs have complex effects on the TME, and in order to further exploit mCRPs as cancer targets, a deeper understanding of how mCRPs impact both the innate and adaptive immune responses is needed. First and foremost, mCRPs act locally in the TME to tightly regulate the activation of the complement cascade at various steps. But more than that, recent data is showing that mCRPs interact with aspects of the adaptive immune response, where by and large, mCRPs are being shown to downregulate T-cell responses to cancer. Generally, this points to an anti-inflammatory role of mCRPs. With mounting evidence that inflammation in the TME is actually beneficial for tumor growth and immune evasion however, it becomes necessary to revisit the role of mCRPs in tumorigenesis and the regulatory mechanisms that may lead to mCRP expression in the first place. What can be established is that mCRP expression in a tumor is overwhelmingly associated with more aggressive TNM staging and, worse overall, patient prognoses. In addition, mCRP expression seems to be specific for tumorigenic tissue and serves as a way to differentiate tumor tissue from adjacent normal tissues. In this review we suggest a new paradigm for understanding mCRP expression in relation to cancer therapy, which is that in the midst of widespread and mounting inflammation within a TME, mCRP expression continually increases as a way to limit pathological complement activation. In doing so, mCRPs become an excellent biomarker for TMEs that are extremely inflammatory, and thus most permissive for aggressive tumor growth and metastasis. In addition to their role as a biomarker, evidence is emerging that neutralizing mAbs against mCRPs can be used to sensitize patients to other chemotherapeutic drugs. Combination therapy of neutralizing mAbs against mCRPs and conventionally using chemotherapy shows great clinical promise. That being said, the role of mCRP expression in cancer is extremely complex and the staging, distribution and intensity of mCRP within the tumor, along with the type of tumor and interactions with combination drugs, need to be taken critically into account when deciding what treatments to use. Finally, it is relatively unknown how mCRPs interact with immunocheckpoint inhibitor therapy, and with the success and widespread use of these therapies, more work needs to be done to elucidate this relationship.

## Author Contributions

AG and JY conceptualized, strategized, and planned the manuscript together. AG wrote and researched the body of the manuscript, and JY advised and edited throughout.

### Conflict of Interest Statement

The authors declare that the research was conducted in the absence of any commercial or financial relationships that could be construed as a potential conflict of interest.
